# CXCL10 Controls Inflammatory Pain via Opioid Peptide-Containing Macrophages in Electroacupuncture

**DOI:** 10.1371/journal.pone.0094696

**Published:** 2014-04-14

**Authors:** Ying Wang, Rebekka Gehringer, Shaaban A. Mousa, Dagmar Hackel, Alexander Brack, Heike L. Rittner

**Affiliations:** 1 Department of Anesthesiology, University Hospital of Würzburg, Würzburg, Germany; 2 Department of Anesthesiology and Critical Care, Charité – Universitätsmedizin Berlin, Campus Virchow-Klinikum, Berlin, Germany

## Abstract

Acupuncture is widely used for pain treatment in patients with osteoarthritis or low back pain, but molecular mechanisms remain largely enigmatic. In the early phase of inflammation neutrophilic chemokines direct opioid-containing neutrophils in the inflamed tissue and stimulate opioid peptide release and antinociception. In this study the molecular pathway and neuroimmune connections in complete Freund's adjuvant (CFA)-induced hind paw inflammation and electroacupuncture for peripheral pain control were analyzed. Free moving Wistar rats with hind paw inflammation were treated twice with electroacupuncture at GB30 (Huan Tiao - gall bladder meridian) (day 0 and 1) and analyzed for mechanical and thermal nociceptive thresholds. The cytokine profiles as well as the expression of opioid peptides were quantified in the inflamed paw. Electroacupuncture elicited long-term antinociception blocked by local injection of anti-opioid peptide antibodies (beta-endorphin, met-enkephalin, dynorphin A). The treatment altered the cytokine profile towards an anti-inflammatory pattern but augmented interferon (IFN)-gamma and the chemokine CXCL10 (IP-10: interferon gamma-inducible protein) protein and mRNA expression with concomitant increased numbers of opioid peptide-containing CXCR3^+^ macrophages. In rats with CFA hind paw inflammation without acupuncture repeated injection of CXCL10 triggered opioid-mediated antinociception and increase opioid-containing macrophages. Conversely, neutralization of CXCL10 time-dependently decreased electroacupuncture-induced antinociception and the number of infiltrating opioid peptide-expressing CXCR3^+^ macrophages. In summary, we describe a novel function of the chemokine CXCL10 - as a regulator for an increase of opioid-containing macrophages and antinociceptive mediator in inflammatory pain and as a key chemokine regulated by electroacupuncture.

## Introduction

Acupuncture has been shown to significantly reduce pain intensity in various pain syndromes e.g. in patients with osteoarthritis [1], low back pain [2] in most, but not all studies [3]. Indeed, due to large cohort studies in patients with low back pain and knee pain this treatment is covered by public heath insurances in some countries including Germany [4]. Despite its widespread use, the underlying mechanisms of acupuncture-induced analgesia are still only incompletely understood. Acupuncture leads to a down-regulation of pro-inflammatory cytokines such as tumor necrosis factor (TNF-alpha) and interleukin (IL)-1beta at the site of inflammation [5], [6]. This anti-inflammatory as well as antinociceptive effect involved activation of the cannabinoid receptor 2 (CB2) [6]. Endogenous opioid peptides such as beta-endorphin (END) also could contribute to acupuncture-induced analgesia. They activate opioid receptors both at the level of the spinal cord [7], [8] as well as on peripheral sensory neurons at the site of inflammation [9], [10]. Acupuncture triggers END transcription and translational in the inflamed tissue and this was attenuated by a CB2 antagonists [11].

Opioid-mediated peripheral antinociception has been extensively studied in models of local hind paw inflammation induced by complete Freund's adjuvant (CFA) [12]. Opioid-containing leukocytes migrate into the inflamed tissue, release opioid peptides such as END, Met-enkephalin (ENK) and dynorphin A (DYN) and induce antinociception by binding to opioid receptors (μ, MOR; δ, DOR and κ, KOR) on peripheral nociceptive neurons. In the early phase of inflammation (first 24 h post induction) neutrophils are the predominant opioid-containing leukocytes whereas monocytes/macrophages are relevant at later stages (>24 h) [13], [14]. Chemokines (CXCL2/3) or corticotrophin releasing hormone can trigger opioid peptide release [14]–[17]. These mediators are either locally injected or they were endogenously released under conditions of stress (cold water swim). The pathophysiological relevance of peripheral opioid-mediated antinociception was more recently demonstrated since bacterial products (formyl peptides) at the site of inflammation bind to formyl peptide receptors on neutrophils leading to tonic release of opioid peptides and a reduced intensity of inflammatory pain [18]. Studies on chemokines have shown that chemokine receptor CXCR2 ligands play a dual role in peripheral antinociception; they are responsible for both the increased numbers of opioid-containing CXCR2^+^ neutrophils to the site of inflammation and the release of opioid peptides from this leukocyte population [16]. In contrast, the role of chemokines at later stages of inflammation when monocytes and macrophages are the major opioid-containing leukocyte population is not well understood. Thus far, the chemokine receptor CCR2 that is expressed on monocytes and peripheral sensory neurons and its ligand CCL2 were shown to act as proalgesic mediators in neuropathic pain and in inflammation [4].

In our study, we explored the molecular mechanisms of peripheral opioid-mediated antinociception in late inflammation and antinociception by electroacupuncture. Specifically, we addressed i) the regulation of cytokines and chemokines by electroacupuncture, ii) the role of the chemokine CXCL10 ( = IP-10, interferon gamma-inducible protein) in CFA inflammation, and iii) the function of CXCL10, opioid-containing macrophages as key regulator of electroacupuncture-induced antinociception.

## Materials and Methods

### Animals and model of inflammation

Animal protocols (REG 69/10) were approved by the governmental animal care committee (Regierung von Unterfranken, Würzburg, Germany) and are in accordance with the International Association for the Study of Pain [19]. Experimental procedures except electroacupuncture treatment were performed under isoflurane anesthesia. Six to ten male Wistar rats (280–350 g) per treatment group were injected intraplantarly (i.pl.) with 150 µl of CFA (Calbiochem, San Diego, CA, USA) in the right hind paw [14].

### Electroacupuncture treatment

A reproducible electroacupuncture protocol in free moving rats performed right after injection of CFA and at 24 h post CFA using 3D image computer modeling was previously established [10]. Rats were randomly divided into CFA+EA (EA) and CFA control (CFA) group and were carefully habituated within the sterilized disposable paper cap three days before experiment. Before needling, the fur above GB30 was shaved on the lower back and disinfected. Briefly, disposable acupuncture needles (Ø = 0.20 mm, length  = 25 mm, schwa-medico, Ehringshausen, Germany) connected to an electrical stimulator (AS Super_4_digital, schwa-medico) were slightly inserted into bilateral GB30 (Huan Tiao). GB30 is widely used to treat sciatica in patients or hind paw pain in rats located on the junction of lateral 2/3 and medial 1/3 on the line between the great trochanter and last sacral vertebrae [20]. The needle position was adjusted if sign of direct irritation of a nerve or blood vessel were noted. The intensity of electroacupuncture was delivered in a gradual and intermittent manner of 2–2.5–3 mA (frequency: 100 Hz, pulse width: 0.1 ms) for 20 min. The exact intensity for different individuals was flexibly kept between 2–3 mA. A slight muscle twitching of the entire hind limb including the paw could be observed as a sign of accurate needling on sciatic nerve underneath of GB30. Rats were kept conscious and allowed for complete free mobilization in the cage during the whole process. For sham treatment needling was performed without application [10].

### Measurement of nociceptive thresholds

Thermal nociceptive thresholds (paw withdrawal latency; PWL) were obtained by the Hargreaves test (IITC Inc/Life Science, Italy) [18]. Rats were habituated in the plastic box with a glass plate underneath for 2–3 d before experiments. The heat of a radiant bulb was adjusted to obtain a paw withdrawal latency of 20 s in the non-inflamed paw. The required time (s) until paw withdrawal was taken as thermal nociceptive threshold. The cut off was set at 30 s to avoid tissue damage. The average of two measurements (with 20 s intervals) was calculated for analysis.

Mechanical nociceptive thresholds (paw pressure threshold; PPT) were evaluated with the paw pressure algesiometer (modified Randall-Selitto test; Ugo Basile, Comerio, Italy) [18]. Rats were habituated into a sterilized disposable man-made cap for several days before experiments and were gently held in the cap during the pain measurements [18]. Increasing pressure (g) was applied to the dorsal surface of paw until the rat withdrew its paw. The cut off point was set at 250 g to avoid tissue damage. Measurements were performed three times (with 10 s intervals) and averages were calculated. All the behavioral tests were performed in a blinded manner.

A value of nociceptive threshold lower than that determined in the contralateral paw usually represents hyperalgesia ( = pain) and values above contralateral thresholds usually represent antinociception ( = analgesia) in animals. Strictly speaking full or partial reversal of hyperalgesia can also be stated as anti-hyperalgesia.

### Pharmacologic interventions

To examine the role of opioid peptides, groups of EA-treated animals were i.pl. injected with anti-opioid peptide antibodies (anti-END, anti-ENK, or anti-DYN; all rabbit anti-rat IgG antibodies, Peninsula, CA, US) at 4 d post CFA induced inflammation. In separate groups of animals, recombinant rat CXCL10 or rabbit anti-rat CXCL10 (both from Peprotech, Hamburg, Germany) was i.pl. administered daily for 5 d (day 0 to 4). Optimal doses were established in preliminary experiments or were based on previous studies [18], [21]. Solvent saline or an identical dose of rabbit IgG was used as a control.

### Enzyme-linked immunosorbent assay (ELISA)

Paw tissue was retrieved at 96 h post CFA injection and minced in ice cold lysis buffer (20 mM imidazole hydrochloride, pH 6.8; 100 mM potassium chloride, 1 mM magnesium chloride, 10 mM ethylene glycol tetraacetic acid, 1.0% Triton X-100, 10 mM sodium fluoride, 1 mM sodium molybdate, 1 mM ethylenediaminetetraacetic acid (Sigma-Aldrich, Munich; Merck, Darmstadt; Carl Roth GmbH, Karlsruhe, all Germany) with complete Protease Inhibitor Cocktail (Roche Diagnostics, Mannheim, Germany). The homogenate was frozen at −80°C. Before experiment, the homogenate was thawed, incubated at 4°C overnight, centrifuged at 14,000 g for 10 min and the supernatant was used for ELISA [13]. ELISA kits were used according to the manufacturer's instructions: IL-1alpha, IL-1beta and interferon (IFN)-gamma (R&D systems, London, UK); CXCL10 (Peprotech, Hamburg, Germany); TNF-alpha and IL-4 (Invitrogen, Life Technologies, Darmstadt, Germany) and IL-13 (Abcam, Cambridge, UK).

### RNA extraction, cDNA transcription and real-time-polymerase chain reaction (RT-PCR)

Rat paw tissues at 72 and 96 h post CFA were homogenized with sterilized stainless steel beads (5 mm, Qiagen, Düsseldorf, Germany) by Tissuelyser (frequency: 20 Hz, Qiagen, Hilden, Germany) [22]. Total RNA was extracted by using TRIzol (Invitrogen/Life Technologies, Carlsbad, CA, USA). Purified RNA (1 µg) was reversely transcribed into cDNA using the High-Capacity cDNA Reverse Transcription Kit. cDNA was diluted 10-fold and amplified by RT-PCR with Taqman gene expression assays for rat CXCL10 (labeled with FAM, Assay ID: Rn01413889_g1) and GAPDH (glyceraldhyde-3- phosphate dehydrogenase, labeled with VIC) as a housekeeping gene (FAM and VIC are compatible fluorescein-based 5′ end reporter dye, the sequence of each primer is confidential from Applied Biosystems/Life Technologies). Assays were performed according to the manufacturer's recommendations using 50 cycles, annealing and extension 1 min at 60°C (7300 System Sequence Detection Software v1.4.0). RT-negative control was applied by all the reagents except the enzyme mix ‘ABsolute QPCR ROX Mix’ (Thermo Fisher Scientific GmbH, Heiligenfeld, Germany) to access the genomic DNA contamination in reverse transcription reaction. Results were calculated using the 2**^ΔΔ^** ^CT^ method for relative quantification. GAPDH was selected as a reference for quantification due to the optimal stable expression in inflamed and non-inflamed paw tissue compared to beta-actin and 18SrRNA in our preliminary experiments (data not shown).

### Immunohistochemistry

Three rats/group at 96 h post CFA were deeply anesthetized with isoflurane and perfused transcardially with 0.1 M phosphate-buffered saline, pH 7.4, and with cold phosphate-buffered saline containing 4% paraformaldehyde pH 7.4 (fixative solution) [23]. The subcutaneous tissue adjacent to the skin was dissected from plantar surfaces of both hind paws, post-fixed in the fixative solution, and cryoprotected in 10% sucrose solution at 4°C overnight, embedded in tissue-Tek compound (OCT, Miles Inc., Elkhart, IN), and frozen. Seven-micrometer-thick sections were prepared on cryostat and mounted on gelatin-coated slides. For double immunostaining, the tissue sections were incubated with a) polyclonal rabbit anti-END or –ENK or –DYN (1∶1000; all from Peninsula Laboratories, Merseyside, UK) in combination with monoclonal mouse anti-CD68 (ED1, 1∶400; Serotec, Düsseldorf, Germany) or b) mouse anti-CXCR3 (1∶500, Biosource, Inc., San Diego, USA) in combination with polyclonal rabbit anti-rat macrophage (1∶200, Cedarlane Laboratories, Ontario Canada). Texas red conjugated goat anti-rabbit antibodies in combination with FITC conjugated donkey anti-mouse antibodies were used as secondary antibodies (all Vector Laboratories, Burlingame, CA). Finally, the tissues were stained with 4′,6 diamidino-2-phenylindole (DAPI) and mounted on vectashield (Vector Laboratories). To demonstrate specificity of staining, omission of the primary antibody was used. The contralateral (contra.) paws without inflammation were stained as negative control (data not shown).

A total of 3 samples from the inflamed paw tissue were imaged in each group, and counting of single- and double-labeled cells was done on confocal images randomly taken from three view fields in each section. Cell counting was performed by a blinded investigator using NIH Image J software (Bethesda, MD, USA). The percentage of double-labeled cells per single-labeled cells was used for statistical analysis.

### Experimental protocols

Before experiments, all animals were randomly divided to CFA+EA, CFA+ sham and CFA control.

1. Antibodies against opioid peptides (END: 2 µg, ENK: 1.25 µg, DYN: 1 µg) were applied (i.pl.) on CFA+EA treated rats at 96 h post CFA. Mechanical and thermal nociceptive threshold changes were assessed 5 min post injection. Control animal received IgG (2 µg).

2. A cytokine array (data not shown) for detecting the relative signals of 29 cytokines/chemokines was performed with paw tissue from CFA or CFA+EA treated rats. ELISA further quantified the protein levels of selected promisingly expressed cytokines/chemokines from cytokine array. CXCL10 was chosen as the targeted chemokine and applied for all subsequent studies due to the most significant upregulation by CFA+EA treatment as well as our former investigations on antinociceptive property of other CXC-chemokines [16].

3. Nociceptive thresholds were daily determined from CFA rats treated with CXCL10 (i.pl., 0.2 ng) or CFA+EA rats treated with the CXCL10 blocking antibody (i.pl., 2 µg) daily from 0–4 d. (a) In selected experiments rats were injected with anti opioid peptide antibodies on day 4 and nociceptive thresholds measured thereafter. (b) Double immunohistochemistry staining on paw tissue sections was conducted for macrophages with either CXCR3 (receptor of CXCL10) or END/ENK/DYN on day 4 after treatment.

### Statistical analysis

All data were presented as mean ± SEM. Data of nociceptive thresholds were given as raw values. Multiple measurements at one time point between two or more than two groups were analyzed by t-test or one way analysis of variance (ANOVA), respectively, e.g. t-test was used for analysis of two groups with one variable factor (e.g. cytokine ELISA from CFA and CFA+EA groups), and one way ANOVA was applied for comparison of multiple groups at one time point (opioid peptide staining from CFA, CFA+EA, CFA+ sham groups). Multiple measurements at different time points between two or more than two groups were analyzed by two way repeated measurement (RM) ANOVA (e.g. all behavioral experiments). Holm-Sidak method was used for one way ANOVA and Student-Newman-Keuls Method was used for two way RM ANOVA. *P<0.05 or **P<0.01 was regarded as statistically significant.

## Results

### Antinociception by electroacupuncture is linked to peripheral opioid peptides

**Figure 1:**
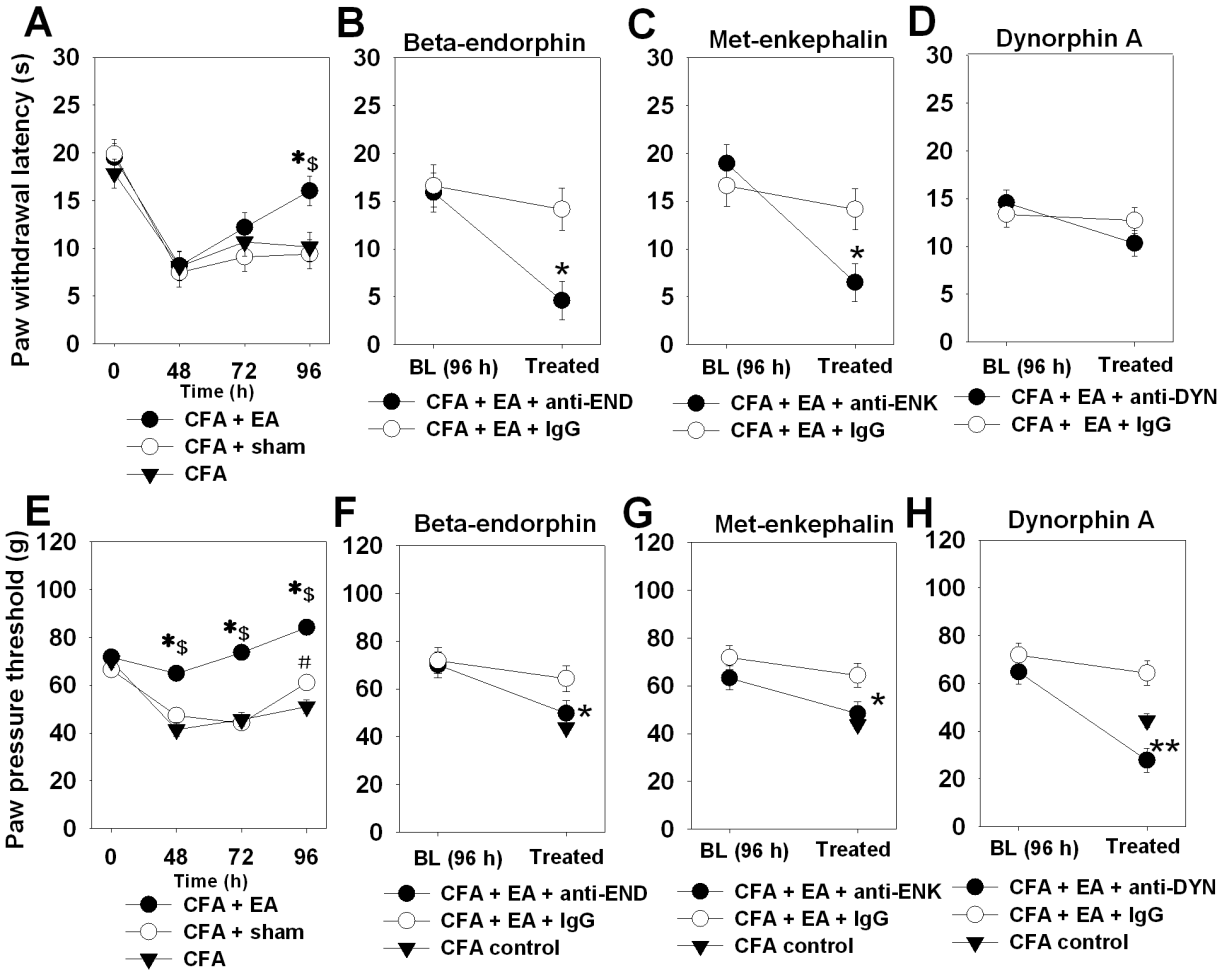
The antinociceptive effect of electroacupuncture (EA) via opioid peptides at the site of inflammation. Wistar rats were injected with CFA i.pl. for 48–96 h and treated with CFA and electroacupuncture (EA) at GB30 at 0 and 24 h (day 0 and 1, 100 Hz, 20 min, 2–3 mA) (CFA+EA). [**A, E**] In previous studies, sham-EA rats did not show significant difference in both mechanical and thermal nociceptive thresholds measurements at 0, 48, 72 and 96 h. Data were presented as mean ± SEM (*p<0.05, CFA+EA versus CFA; ^$^p<0.05, CFA+EA versus CFA+ sham; ^#^p<0.05, CFA+ sham versus CFA; Two-way RM ANOVA, Student-Newman-Keuls). We therefore omitted sham-EA treatment in following studies. Anti-END (2 µg [**B, F**], anti-ENK (1.25 µg [**C, G**]) or anti-DYN (1 µg [**D, H**]) was locally injected (i.pl.) at 4 d post CFA and concomitant twice EA treatment (black circles). Two control groups were added: injection with identical doses of nonspecific anti-rabbit IgG (white circle) or for comparison CFA without EA (black triangle). Paw withdrawal latency (thermal nociceptive thresholds [**A–D**]) or paw pressure thresholds (mechanical nociceptive thresholds [**E–H**]) were determined before (BL: baseline) and 5 min after injection (treated). All the data are presented as mean ± SEM (n = 6 per group, *p<0.05, **p<0.01, CFA+EA+IgG versus CFA+EA+anti-END/ENK/DYN; Two-way RM ANOVA, Student-Newman-Keuls).

In a previous study, at 96 h CFA, electroacupuncture at GB30 caused antinociception in CFA inflammation which was fully blocked by peripheral injection (i.pl.) of the opioid receptor antagonist naloxone at the site of inflammation [10]. Sham-EA treatment (needling without application of current) did not elicit a comparable antinociceptive effect in both mechanical and thermal nociceptive threshold tests (**Fig. 1A, E**). Local injection of antibodies against the opioid peptides END or ENK significantly inhibited electroacupuncture-mediated mechanical and thermal antinociception at 5 min post injection compared to isotype control antibody (**Fig. 1B, C, F, G**, doses according to [18]). There was no significant difference between CFA baseline paw pressure threshold at 96 h and CFA+EA and anti-END or anti-DYN paw pressure thresholds. Anti-END or anti-ENK injection in CFA+EA rats thermal thresholds even more than baseline CFA levels at 96 h. Simultaneous injection of anti-END and anti-ENK antibodies did not cause an additive effect (data not shown). Antibodies against DYN (i.pl., doses according to [21]) completely blocked antinociception to mechanical (**Fig. 1H**) but not thermal stimuli (**Fig. 1D**). Nociceptive thresholds of non-inflamed paws from the same experiments in **Fig. 1** were not significantly altered as displayed in **Fig. S1**, manifesting the peripheral other than the central opioid peptide-related mechanism involved in the study. Due to the more pronounced effects of EA on mechanical nociceptive thresholds and different mechanisms of thermal and mechanical hyperalgesia we focused on these in subsequent experiments.

### Electroacupuncture regulates expression of certain cytokines in the inflamed paw

**Figure 2:**
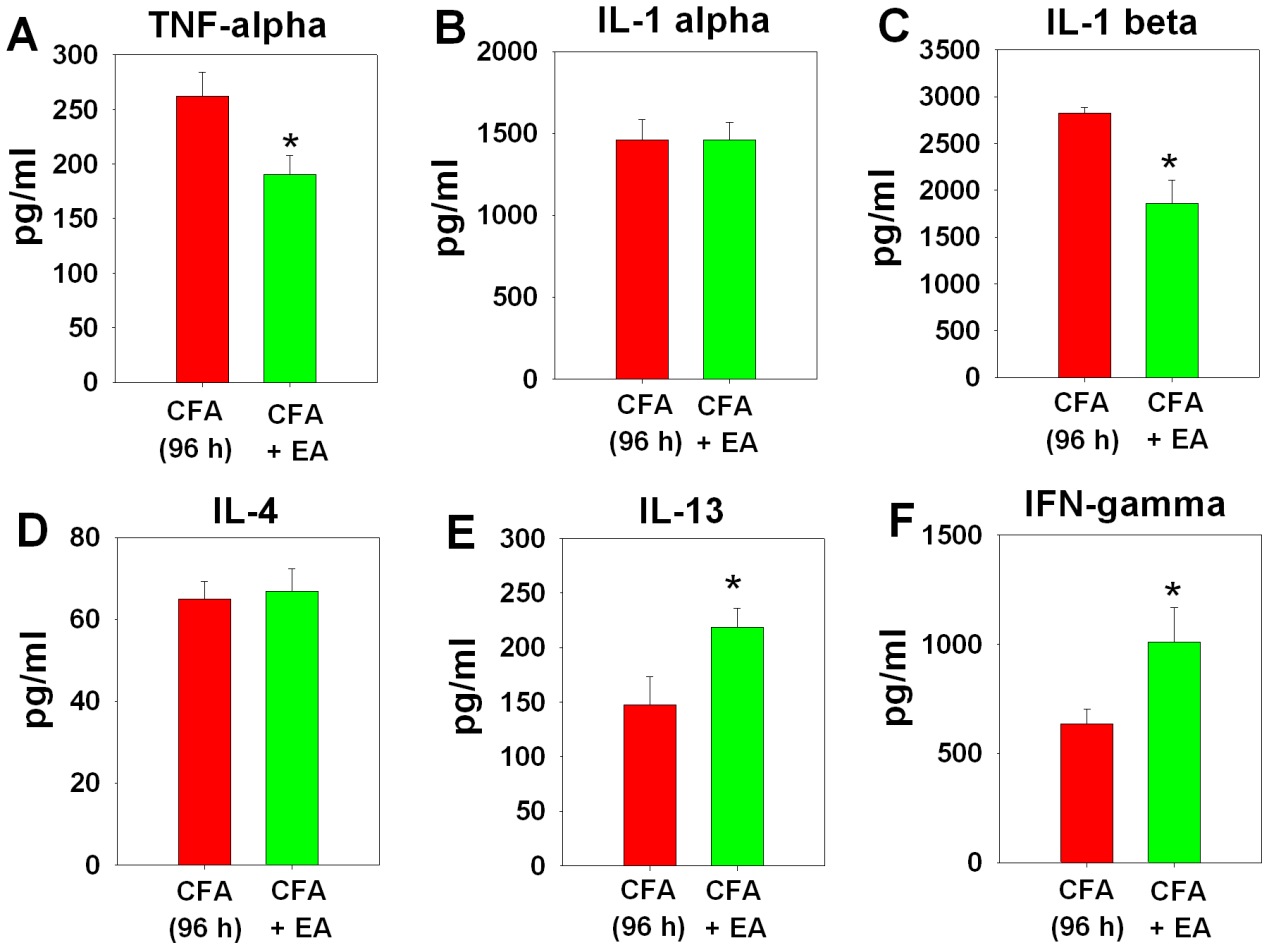
Differential alterations in pro- and anti-inflammatory cytokines in inflamed paw tissue by electroacupuncture (EA). Rats were injected with CFA treated with (CFA+EA) or without (CFA) EA. Based on the results from pilot experiments for immune array of 29 cytokines (data not shown), [**A–F**] *pro- and anti-inflammatory* cytokines including TNF-alpha, IL-1alpha, IL-1beta, IFN-gamma, IL-4 and IL-13 in the paws were selectively quantified by ELISA after 96 h CFA. Data are presented as mean ± SEM (n = 5–10 per group, *p<0.05, CFA+EA versus CFA; t-test).

At 96 h CFA, based on a cytokine array detecting a total 29 cytokines (data not shown), we selectively quantified the protein level of several positive cytokines. Pro-inflammatory cytokines including TNF-alpha and IL-1beta (**Fig. 2A, C**) were significantly downregulated by electroacupuncture whereas IL-1alpha (**Fig. 2B**) was unaltered. The anti-inflammatory cytokine IL-4 remained unchanged whereas IL-13 was significantly upregulated (**Fig. 2D, E**). Interestingly, the only pro-inflammatory cytokine that was significantly upregulated was IFN-gamma (**Fig. 2F**).

### CXCL10 expression and increased numbers of opioid-containing macrophages are associated with electroacupuncture

**Figure 3:**
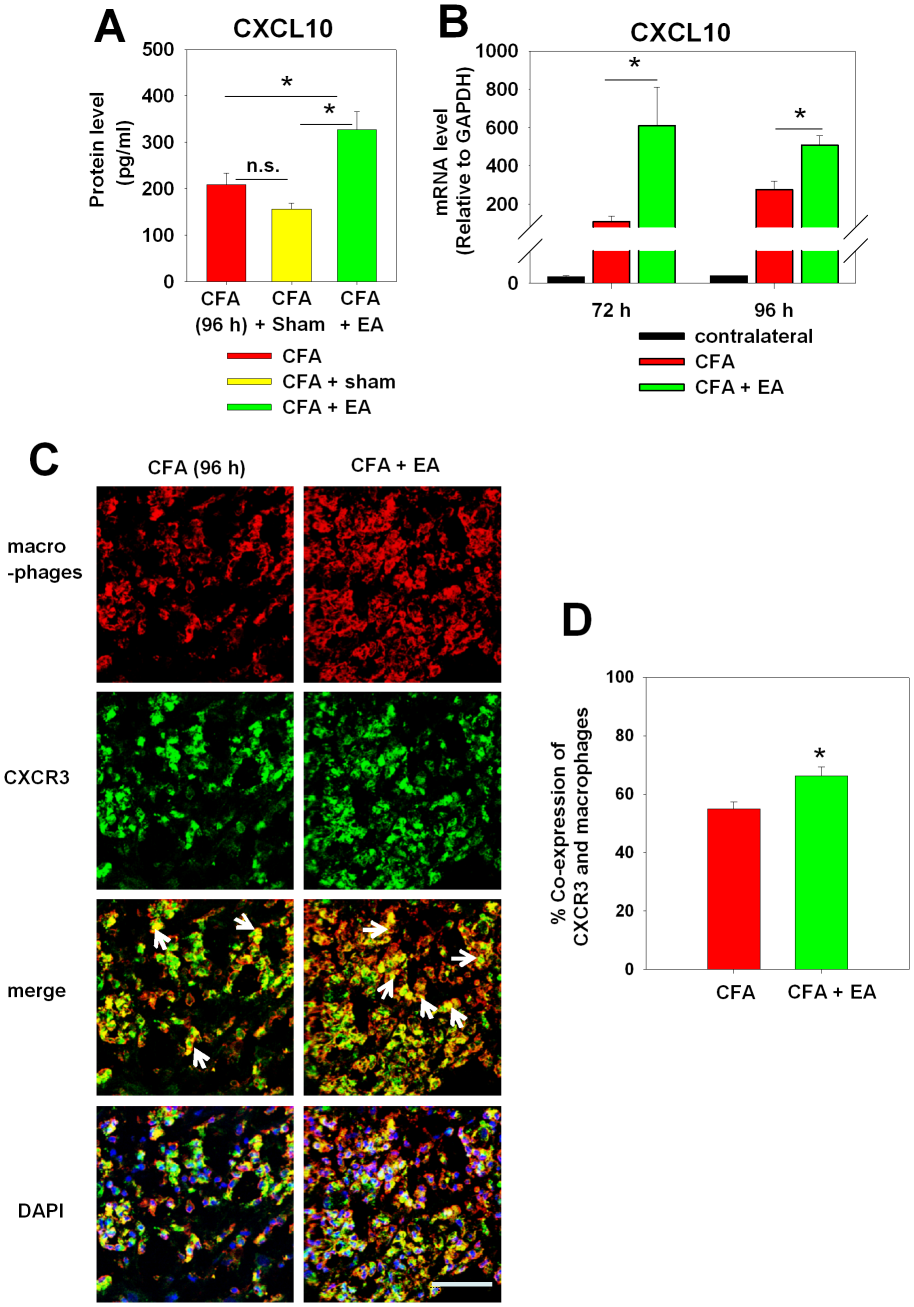
Upregulation of CXCL10 and an increase of CXCR3^+^macrophages in inflamed paw tissue by electroacupuncture (EA). Rats were injected with CFA and treated with (CFA+EA), (CFA+sham) or CFA only. On day 4 (96 h), CXCL10 was quantified by ELISA [**A**] and semi-quantitative RT-PCR (72 and 96 h) in subcutaneous paw tissue ([**B**] noninflamed contralateral paw (contra.) is only shown as a negative control). Data are presented as mean ± SEM (For ELISA: n = 6 per group, *p<0.05, one way ANOVA, Holm-Sidak method; For RT-PCR: n = 6 per group, *p<0.05, CFA+EA versus CFA; t-test). [**C**] Tissue sections were stained with rabbit anti-rat macrophage serum (red), mouse anti-rat CXCR3 antibody (green) and DAPI. The arrows are pointing at CXCR3 expressed macrophages. Representative sections are shown, arrows pointing on double positive cells (scale bar: 50 µm). [**D**] The percentage of macrophages and opioid positive cells was analyzed. All data are presented as mean ± SEM (n = 3 per group, *p<0.05, CFA+EA versus CFA; t-test).

CXCL10 is a chemokine stimulated by IFN-gamma [24]. Since IFN-gamma was the only pro-inflammatory cytokine upregulated by electroacupuncture, we focused our subsequent experiments on CXCL10. Electroacupuncture significantly upregulated CXCL10 on both the protein (**Fig. 3A**) and mRNA level (**Fig. 3B**). No CXCL10 protein increase was seen in sham-treated animals.

Neutrophils are an important source of opioid peptides released in the early phase of CFA-inflammation (up to 24 h) whereas macrophages are considered mainly responsible for peripheral opioid peptide-mediated antinociception at later stage of inflammation (48–96 h). T cells constitute only a small subpopulation of infiltrating leukocytes (<5%) [14]. Since CXCL10 exclusively binds to the chemokine receptor CXCR3 [25], we analyzed the co-expression of CXCR3 with a rabbit anti-rat macrophage serum at the site of inflammation with or without concomitant electroacupuncture. CXCR3 was expressed on the vast majority of infiltrating macrophages (**Fig. 3C**). Furthermore, the percentage of CXCR3^+^ expressing macrophages was significantly increased by electroacupuncture (**Fig. 3D**).

### CXCL10 reverses CFA-induced mechanical hyperalgesia via peripheral opioid peptides

**Figure 4:**
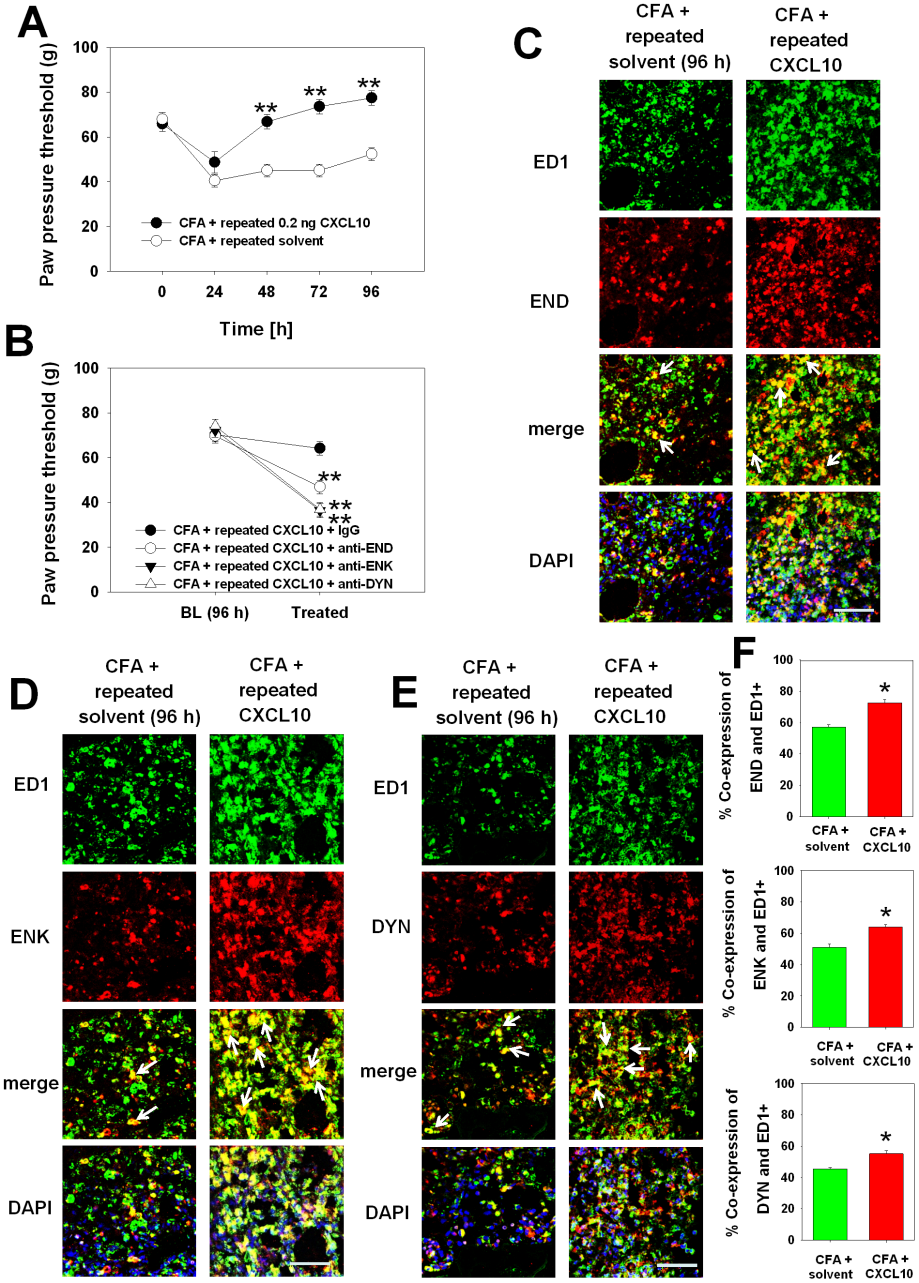
Opioid peptide–dependent sustained antinociception and increase opioid peptide expressed macrophages by repeated CXCL10 injection. Rats were i.pl. injected with CFA and daily with CXCL10 (0.2 ng) or solvent control. [A] Mechanical nociceptive thresholds were determined daily before each injection. Data were presented as mean ± SEM (n = 6 per group, *p<0.05, **p<0.01, CFA+CXCL10 versus CFA+solvent; Two-way RM ANOVA, Student-Newman-Keuls). [B] Anti-END (2 µg, anti-ENK (1.25 µg) or anti-DYN (1 µg) was locally injected (i.pl.) at 4 d post CFA on rats with repeated injection of CXCL10 (0.2 ng). Identical doses of anti-rabbit IgG were used as control. Data were presented as mean ± SEM (n = 6 per group, *p<0.05, **p<0.01, CFA+CXCL10+IgG versus CFA+CXCL10+anti-END/ENK/DYN; Two-way RM ANOVA, Student-Newman-Keuls). [C] Immunohistochemical staining of paw tissue was performed at 96 h with a mouse anti-ED1 (CD68) macrophage antibody (green) and with rabbit anti-END, anti-ENK or anti-DYN antibodies (all was marked red) as well as DAPI. Representative sections are shown. Arrows pointing at double positive cells. (scale bar: 50 µm). [D] The percentage of END/ENK/DYN+ and ED1+ was quantified. All the data are presented as mean ± SEM (n = 3 per group, *p<0.05, CFA+ solvent versus CFA+CXCL10; t-test).

Chemokines like CXCL1 and CXCL2/3 play a dual role in peripheral opioid peptide mediated antinociception, because they recruit opioid-containing neutrophils and trigger opioid peptide release via its receptor CXCR2 [16]. To address whether CXCL10 was able to attenuate inflammatory pain by CFA via increased numbers of opioid containing cells we performed multiple injection of CXCL10. Repeated daily administration of 0.2 ng CXCL10 (based on a preliminary dose-finding study, data not shown) elicited sustained mechanical antinociception at 48, 72 and 96 h (**Fig. 4A**). CXCL10-mediated antinociception was fully reversed by the concomitant i.pl. administration of CXCL10 with anti-END, anti-ENK, or anti-DYN at 96 h (**Fig. 4B**). In parallel, repeated chemokine injection was associated with a significant increase in ED1^+^ macrophages co-expressing the opioid peptides END, ENK, and DYN in comparison to solvent control (**Fig. 4C–E**). More ED1^+^ macrophages expressed ENK (almost 72%) compared to END (63%) and DYN (55%) (**Fig. 4F**). No change was seen in rats treated with solvent.

### Electroacupuncture increases ligand availability

**Figure 5:**
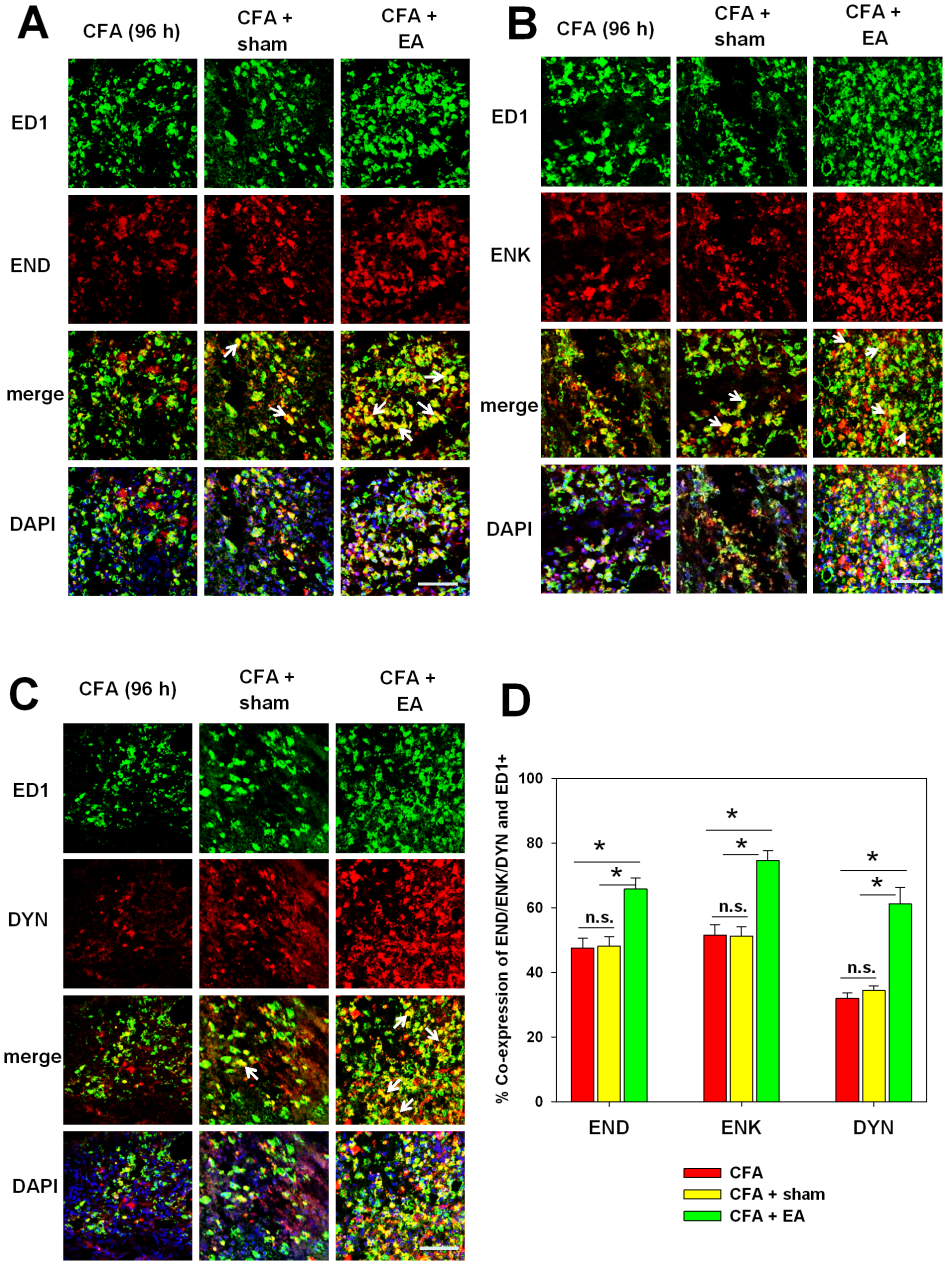
EA enhanced the recruitment of opioid-containing macrophages. Rats were injected with CFA with (CFA+EA), (CFA + sham) or without (CFA) EA treatment for 4 days. Immunohistochemical staining was performed for mouse anti-CD68 macrophages (green) and rabbit [**A**] anti-END, [**B**] anti-ENK or [**C**] anti-DYN antibodies respectively (red). DAPI (blue) was used to recognize cell nuclei (Representative sections are shown by arrows, scale bars: 50 µm). [**D**] The percentage of ED1 and opioid positive cells was quantified. All the data are presented as mean ± SEM (n = 3 per group, *p<0.05, one way ANOVA, Holm-Sidak method).

Next, we tested whether electroacupuncture affects the accumulation of opioid peptide. Electroacupuncture was associated with a significant increase in the number of ED1^+^ macrophages co-expressing the three opioid peptides END, ENK and DYN (**Fig. 5A, B, C**). More ED1^+^ macrophages expressed ENK (almost 75%) compared to END (65%) and DYN (60%) (**Fig. 5D**). No change on the co-expression of opioid peptides and ED1 was seen in sham treated rats.

### Electroacupuncture-induced antinociception and increased numbers of opioid-containing macrophages is prevented by blockade of CXCL10

**Figure 6:**
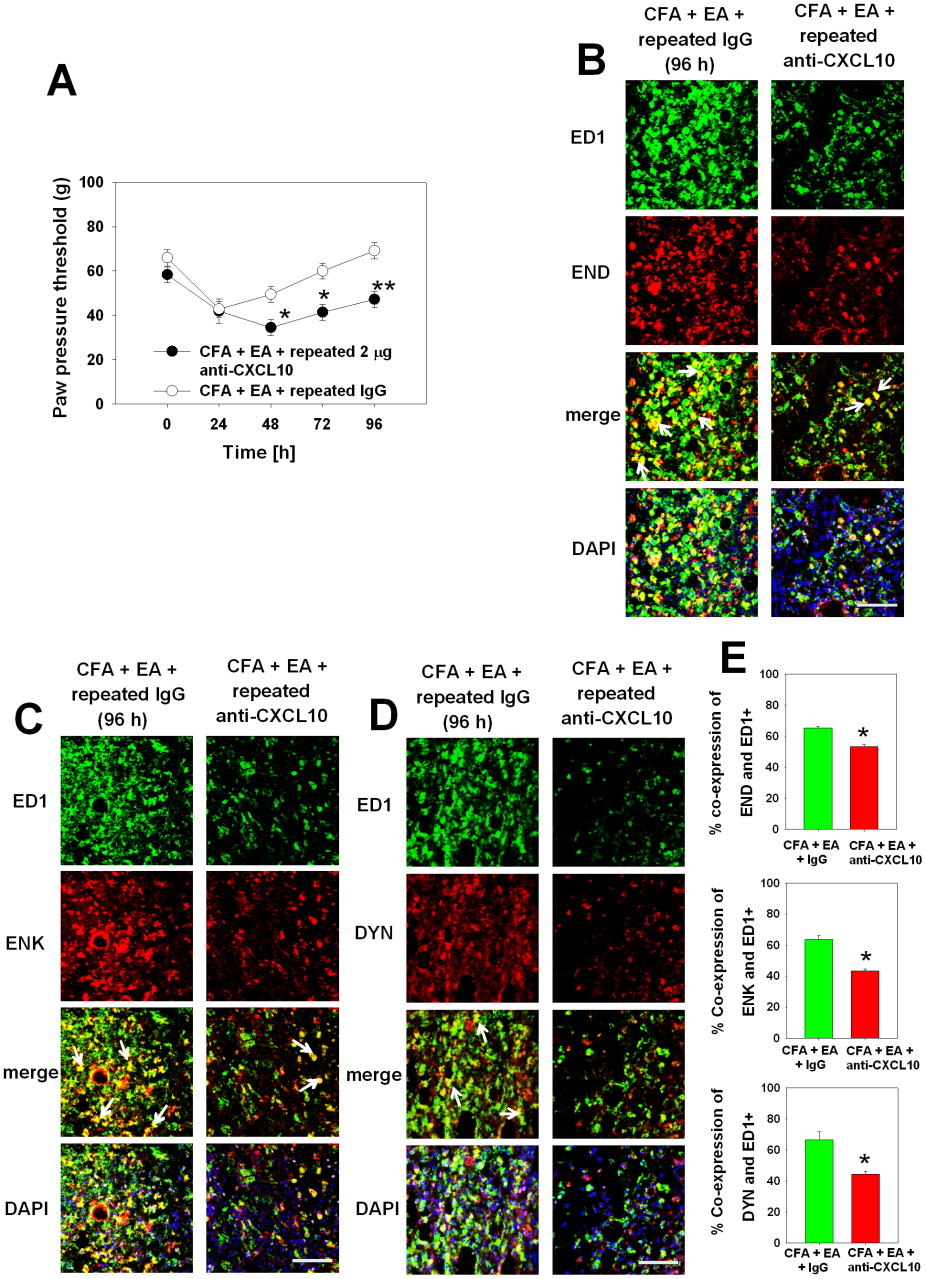
Neutralization of CXCL10 fully reversed electroacupuncture (EA)-induced antinociception and increase of opioid-containing monocytes/macrophages. [A] Rats with CFA inflammation and EA treatment were daily i.pl. injected with an antibody against CXCL10. Controls were injected with anti-rabbit IgG antibody. Mechanical nociceptive thresholds were determined before (BL) and after injections. Data are presented as mean ± SEM (n = 6 per group, *p<0.05, **p<0.01, CFA+EA+IgG versus CFA+EA+anti-CXCL10; Two-way RM ANOVA, Student-Newman-Keuls). [B–D] Immunohistochemical staining was performed for mouse anti-ED1 monocytes/macrophages (green) and rabbit anti-END, anti-ENK or anti-DYN antibodies respectively (red). DAPI (blue) was used to recognize cell nuclei. Representative sections are shown, arrows pointing at double positive cells (scale bar: 50 µm). [E] Quantification for immunohistochemical staining showed the percentage of double positive ED1 and END/ENK/DYN cells. All the data are presented as mean ± SEM (n = 3 per group, *p<0.05, CFA+EA+IgG versus CFA+EA+anti-CXCL10; t-test).

Since electroacupuncture-elicited antinociception correlated with the number of opioid-containing macrophages, we examined whether CXCL10 was a key regulator. Daily injections of anti-CXCL10 significantly decreased the pain threshold at 48–96 h post CFA and abolished the electroacupuncture-induced antinociception (**Fig. 6A**). Furthermore, multiple injections of the anti-CXCL10 significantly reduced the number of ED1^+^ macrophages co-expressing the opioid peptides END, ENK, and DYN (**Fig. 6B, C, D**) stimulated by electroacupuncture. The percentage of ED1^+^ macrophages expressed END was reduced to 53% compared to ENK (43%) and DYN (44%) (**Fig. 6E**). No change was seen in rats treated with isotype control antibody.

## Discussion

Acupuncture is widely used as an alternative analgesic therapy in a broad range of pain syndromes. Despite its widespread use the role is often doubted and attributed to placebo effects. Indeed, the underlying molecular mechanisms of pain control are not well understood. In our study electroacupuncture suppressed selected pro- and enhanced anti-inflammatory cytokines in a model of inflammatory pain in rats. In contrast to this pattern, EA increased the production of the cytokine IFN-gamma and the chemokine CXCL10 at the site of inflammation leading to an increase in opioid-containing CXCR3^+^ macrophages. Macrophage-derived opioid peptides could activate opioid receptors on peripheral sensory neurons and suppressed inflammatory pain. Taken together we identified a new molecular pathway of acupuncture-induced analgesia.

### Effects of electroacupuncture on the peripheral opioid system and on cytokine production

Endogenous opioid peptides inhibit pain both in the central nervous system and in the periphery. Acupuncture triggers the release of opioid peptides at the level of the spinal cord and in the brain leading to activation of MOR and sometimes other opioid receptors [7], [26]–[29]. In the periphery, opioid peptide-induced antinociception seems to involve MOR, DOR and KOR at the site of inflammation [9], [10], [30]. There are some hints that acupuncture regulates the expression of the END in immune cells and in keratinocytes via activation of the cannabinoid receptor CB2 [11]. We now extended these findings by demonstrating that electroacupuncture stimulated the increased numbers of leukocytes containing the three opioid peptides END, ENK, and DYN and that all three opioid peptides mediated antinociception to thermal and mechanical stimuli (similar to stress (cold water swim)-induced antinociception [12]). In our study, DYN did not contribute to thermal antinociception induced by acupuncture. Anti-END and anti-ENK treatment even lowered thermal nociceptive thresholds more than CFA baseline at 96 h possibly due to endogenous tonic release of opioid peptides in thermal hyperalgesia [18]. In accordance with our study here, other groups also observed that electroacupuncture better controls mechanical than thermal hyperalgesia in inflammatory pain [31], probably due to different mechanisms and receptors controlling thermal and mechanical pain.

In addition to the upregulation of opioid peptides, acupuncture was previously claimed to suppress the production of the pro-inflammatory cytokines TNF-alpha, IL-1beta and IL-6 in inflammatory pain [6], [11]. Studies in other models (asthma, trauma) demonstrated that T_h_1 cytokines such as IL-2 and IFN-gamma were increased whereas T_h_2 cytokines such as IL-4, IL-10 and IL-13 were suppressed [32]–[34]. In our model of inflammatory pain, electroacupuncture significantly attenuated IL-1beta and TNF-alpha as well as selectively upregulated the T_h_1 cell type cytokine IFN-gamma. In contrast to the findings in models of asthma and trauma, the T_h_2 cytokine IL-4 was unchanged and IL-13 was upregulated indicating the activation of both T_h_1 and T_h_2 cell signaling by electroacupuncture. Therefore, differential effects of EA on cytokines are observed in different models. Despite the suppression of pro-inflammatory cytokines we and others [6] found that the number of infiltrating opioid peptide-containing leukocytes (i.e. macrophages) was significantly increased by electroacupuncture. In line with our previous studies [13], [14] T cells were almost absent at the site of CFA-induced inflammation (data not shown). In summary, our study in part supports the previously described anti-inflammatory effects of electroacupuncture, but some pro-inflammatory cytokines like IFN-gamma and CXCL10 seem to be upregulated in inflammatory pain.

### Chemokines in inflammation and nociception

In our model of inflammatory pain, the expression of the chemokine CXCL10 and, in parallel, the numbers of macrophages expressing the corresponding chemokine receptor CXCR3 were significantly upregulated by electroacupuncture. Few studies examined the role of electroacupuncture on chemokine expression. It was reported electroacupuncture augmented the production of the chemokine CXCL12 (stromal cell-derived factor-1alpha) in cerebral ischemic injury [35] whereas the production of CCL2 (monocyte chemotactic protein-1) was downregulated in adipose tissue without any accompanying inflammation [36]. While these studies focused on acupuncture, the role of chemokines in hyperalgesia and antinociception has been studied on more general level. The monocytic chemokine CCL2 and its corresponding receptor CCR2 were shown to be proalgesic mediators in neuropathic and other pain models [37]. Interestingly, chemokine receptors (including CCR2 and CXCR4) could interact with receptors involved in antinociception (MOR) or inflammation (adenosine A2A receptor). Activation of one receptor leads to the trans-deactivation of the other. Crosstalk between chemokines and neuronal receptors bridges immune and nervous systems [38]. Studies examining the chemokine-mediated selective recruitment of isolated leukocyte subpopulations to non-inflamed skin demonstrated that the monocytic chemokine CCL2 and the neutrophilic chemokine CXCL1 or CXCL2/3 induced recruitment of the respective leukocyte population, but while nociceptive thresholds were unchanged by CXCL2/3 [39], CCL2 elicited hyperalgesia [23], [40]. The role of CXCL10 in pain is not very well examined. Toll like receptor ligands can induce expression and production of pro-inflammatory chemokines and cytokines including CXCL10 or e.g. IL-1alpha, IL-1beta, and PGE_2_ in dorsal root ganglia neurons, which in part have previously been shown to increase pain [41]. In summary, the role of cytokines and chemokines in the generation of hyperalgesia or antinociception depends on the model and the state of inflammation.

### The broad spectrum of CXCL10-mediated actions in inflammation

Electroacupuncture augmented the CXCL10 expression both on the transcriptional and translational level and increased the number of opioid containing CXCR3^+^ macrophages as well as long-lasting antinociception. Repeated injections of CXCL10 reversed hyperalgesia in CFA rats. Similarly, repeated anti-CXCL10 in EA-treated animals lessened the antinociceptive effect of EA. CXCL10 is upregulated by IFN-gamma, which was also increased after treatment with electroacupuncture. CXCL10 is a chemoattractant for activated T cells, monocytes/macrophages, dendritic cells and microglia [24]. In addition to recruiting inflammatory cells, CXCL10 induced astroglial proliferation and is directly neurotoxic e.g. in the HIV-1 neuropathogenesis [42]. Interestingly, the CXCL10/CXCR3 interaction played an important role in tuberculosis. Both tuberculosis and CFA-induced hind paw inflammation are caused by different strains of mycobacteria. CXCL10 production was upregulated in macrophages (and to a lesser degree in dendritic cells) by mycobacterium tuberculosis *in vitro* and *in vivo* [43]. This is a hallmark of active – but not latent – infection [44] similar to our study of CFA inflammation with heat killed and dried mycobacteria in oily solution as a nonspecific active inflammation. Furthermore, CXCL10 regulates the recruitment of CXCR3^+^ macrophages to the vessel wall [45]. In contrast in our CFA model, macrophages were the predominant leukocyte population, T cells were virtually absent (<5%) and NK cells have thus far not been studied [13], [14]. Thus, CXCL10 seemed to preferentially interact with opioid-containing CXCR3^+^ macrophages.

### The novel role of CXCL10 – a key regulator of antinociception in acupuncture

Repeated daily injection of CXCL10 conferred sustained antinociception and lead to a parallel increase in the number of opioid peptide-expressing macrophages. Our data favor the hypothesis that electroacupuncture influenced the transcription and translation of CXCL10. How could this be mediated? One obvious candidate would be adenosine since manual acupuncture triggers its release and antinociception is mediated by adenosine A1 receptors [46]. However, adenosine receptor activation decreased, rather than increased CXCL10 production in macrophages [47]. Alternatively, cannabinoid receptors could be involved since they contributed to acupuncture-induced antinociception [5], [6]. However, they also suppressed inflammation and downregulated chemokines at least in keratinocytes [48]. Although the immune regulation of peripheral acupuncture-induced antinociception appears well understood, the molecular link between the peripheral nervous system (presumably activated by acupuncture) and the immune regulation remains enigmatic to be solved.

### Availability of opioid receptors and ligands for pain control

Previously, expansion of opioid-containing neutrophils by hematopoietic factors [22] or enhanced recruitment by local injection of neutrophilic chemokines [49] did not enhance peripheral opioid-mediated antinociception in early stages of inflammation since opioid receptor expression was limiting antinociception. In addition electroacupuncture did not further alter the expression of opioid receptors in the inflamed paw (data not shown). In contrast to the findings of expansion or recruitment of opioid-containing neutrophils, enhanced recruitment of opioid-containing macrophages induced antinociception in later stages of inflammation probably because the opioid receptors are already upregulated after 4 d of inflammation and not fully occupied by the available ligands. Importantly, neutralization by an anti-CXCL10 antibody time-dependently inhibited electroacupuncture-elicited antinociception by daily injection. It also largely suppressed the increased numbers of opioid-containing macrophages. In summary, intensity of antinociception is regulated differently during inflammation. Antinociception is limited by opioid receptor availability in early and ligand availability (i.e. opioid peptides) in late inflammation. Accordingly, increased numbers of opioid-containing leukocytes enhance antinociception in late but not in early inflammation.

Taken together, our data suggest that electroacupuncture enhances CXCL10 production at the site of inflammation and stimulates peripheral opioid peptide-mediated antinociception. Furthermore CXCL10 itself appears to trigger an increased number of opioid-containing monocytes/macrophages at the site of inflammation without acupuncture. Our data suggest that CXCL10 appears to be a key antinociceptive mediator also in electroacupuncture-mediated analgesia.

## Supporting Information

Figure S1 **[A–H] Nociceptive thresholds of non-inflamed paws in Fig. S1 were measured as contralateral controls.** No statistical difference was observed between each group at given time points. All the data are presented as mean ± SEM (n = 6 per group, two way RM ANOVA).
(TIF)
