# Correction: CXCL10 Controls Inflammatory Pain via Opioid Peptide-Containing Macrophages in Electroacupuncture

**DOI:** 10.1371/journal.pone.0105841

**Published:** 2014-08-08

**Authors:** The PLOS ONE Staff

There were additional funders for this article that were not included previously. Please refer to the correct funding statement below:

This study was supported by a grant of the German medical acupuncture association (DÄgfA), China scholarship council and University Funds from the University Hospitals of Wuerzburg. This publication was funded by the German Research Foundation (DFG) and the University of Wuerzburg in the funding program Open Access Publishing. The funders had no role in study design, data collection and analysis, decision to publish, or preparation of the manuscript.
