# Supplementary figures and images for: CXCL10 Controls Inflammatory Pain via Opioid Peptide-Containing Macrophages in Electroacupuncture
Source: PLoS One. 2014 Apr 14;9(4):e94696. doi: 10.1371/journal.pone.0094696 (PMC3986408; doi:10.1371/journal.pone.0094696)

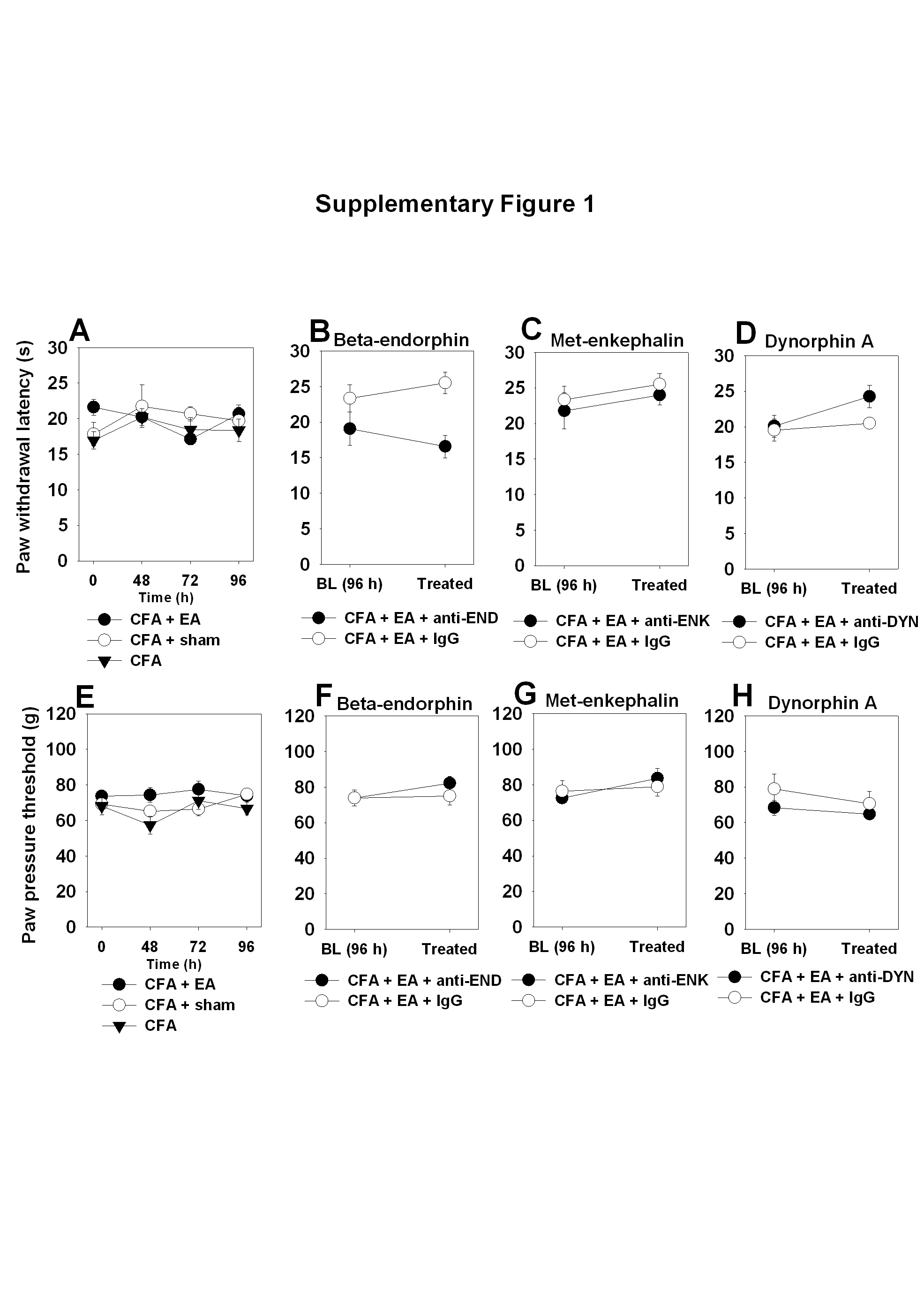

Supplement: Figure S1 — [A–H] Nociceptive thresholds of non-inflamed paws in Fig. S1 were measured as contralateral controls. No statistical difference was observed between each group at given time points. All the data are presented as mean ± SEM (n = 6 per group, two way RM ANOVA). (TIF) [file pone.0094696.s001.tif]
